# Spontaneous charged lipid transfer between lipid vesicles

**DOI:** 10.1038/s41598-017-12611-0

**Published:** 2017-10-03

**Authors:** Joanna L. Richens, Arwen I. I. Tyler, Hanna M. G. Barriga, Jonathan P. Bramble, Robert V. Law, Nicholas J. Brooks, John M. Seddon, Oscar Ces, Paul O’Shea

**Affiliations:** 1 School of Life Sciences, University of Nottingham, Nottingham, United Kingdom; 2 Food Colloids and Processing Group, School of Food Science and Nutrition, University of Leeds, Leeds, LS2 9JT United Kingdom; 3 Department of Medical Biochemistry and Biophysics, Karolinska Institutet, Stockholm, Sweden; 4 Department of Chemistry, Imperial College London, South Kensington, London, SW7 2AZ United Kingdom; 5 Faculty of Pharmaceutical Sciences, University of British Columbia, Vancouver, V6T 1Z3 Canada

## Abstract

An assay to study the spontaneous charged lipid transfer between lipid vesicles is described. A donor/acceptor vesicle system is employed, where neutrally charged acceptor vesicles are fluorescently labelled with the electrostatic membrane probe Fluoresceinphosphatidylethanolamine (FPE). Upon addition of charged donor vesicles, transfer of negatively charged lipid occurs, resulting in a fluorescently detectable change in the membrane potential of the acceptor vesicles. Using this approach we have studied the transfer properties of a range of lipids, varying both the headgroup and the chain length. At the low vesicle concentrations chosen, the transfer follows a first-order process where lipid monomers are transferred presumably through the aqueous solution phase from donor to acceptor vesicle. The rate of transfer decreases with increasing chain length which is consistent with energy models previously reported for lipid monomer vesicle interactions. Our assay improves on existing methods allowing the study of a range of unmodified lipids, continuous monitoring of transfer and simplified experimental procedures.

## Introduction

The lipidic component of cells is extremely diverse comprising thousands of different molecules broadly classified into eight subtypes which include fatty acyls, glycerophospholipids, sphingolipids and sterols^1^. Functionally, lipids have three general purposes: to provide energy storage, to form membrane structures which provide structure and compartmentalisation within the cellular environment and to act as messengers in signal transduction and molecular recognition processes^2^. Within a cellular membrane lipids provide the capacity for processes such as budding and fusion which in turn are fundamental to many essential cellular functions including cell division and intracellular membrane trafficking^3^.

Whilst technological developments are enabling the identification of the many diverse lipidic species in existence, our understanding of why cells devote precious resources to a large diversity of specific synthesis remains unclear^4^. One contributing factor to this is the experimental difficulty encountered when trying to discriminate mechanisms of action and properties which can be attributed to the different species. Studies investigating the properties of different acyl chains, for example, are hindered by their hydrophobic nature which limits many of their properties to be measured as a continuum phase rather than as individual molecules^5^.

Lipid dynamics, movement and transport are crucial for facilitating the multitude of functional properties attributed to lipids and for maintaining the diversity of lipid compositions found within different organelles^4^. It is coordinated by a number of mechanisms including vesicular transport, protein-mediated movement, lateral diffusion and transbilayer flip-flop^6–8^. Spontaneous lipid transport (SLT) is a mechanism whereby lipid molecules move between membranes without any catalytic (i.e. enzymic/protein) assistance via aqueous diffusion, collision or activated collision based systems^7^. There is a view that it has limited biological relevance due to both the perceived slow rate at which the process occurs and potential incompatibility with the lipid compositional gradients observed to exist between organelles^6,8^. It is apparent, however, that the rate at which SLT occurs is dependent on several factors including bilayer composition, vesicle curvature and vesicle concentration^2,8–11^. Similarly the measured rate of transfer^12^ varies with the nature of the lipid under consideration particularly if they are modified by molecular probes, from the order of seconds to the order of days^7^. This in view of all these considerations it is feasible that the importance of SLT within a cellular environment has been underestimated.

Previous studies have used a number of experimental methods to characterise the transfer rates and thermodynamic properties of SLT, which include observing changes in pyrene excimer formation^13^, quantification of fluorescently labelled lipid transfer^14^, resonant energy transfer between fluorescently labelled lipids^15^ and the separation of radiolabelled lipid vesicles after transfer^16–19^. The most widely accepted concept used to describe spontaneous lipid transfer is the lipid monomer diffusion model. In this model, lipids are desorbed from a vesicle or bilayer and diffuse through the aqueous phase until they are absorbed by another vesicle or bilayer. The kinetics of this process appear to be dominated by the low rate of desorption of a lipid from a vesicle into aqueous solution.

From a number of studies^13,15,19^ a thermodynamic model has been developed which suggests that the lipid must be in a thermally driven high energy transition state prior to desorption from the vesicle surface. The activation enthalpy increases with acyl chain length and the free energy for absorption of a free lipid decreases. So we would expect to observe slower rates of transfer for PC lipids with longer chains.

Further studies^18,20^ undertaken at higher lipid concentrations indicate that additional terms for the vesicle concentration must be included in the model to account for the intervesicular collisions. At vesicle concentrations below 2 mM lipid (of vesicles of a similar diameter and thus includes the results presented in this study) the influence of such collisions are not experimentally observed^18,20^. Note that this contribution was in addition to the aqueous phase transport, rather than an explanation of the transfer rates observed. It has been shown with a mathematical analysis of lipid transfer in vesicle systems by Almedia^21^ that the dominant effect in determining the transfer characteristics remains to be the high energy required to desorb the lipid from the vesicle. It was shown that for asymmetric systems, where there are many more acceptor vesicles than donors, the statistical effect of high acceptor concentrations (and shorter acceptor-donor distance) is insufficient to explain the increased transfer rates shown experimentally. Therefore the effect could be due to collisional processes where the donor lipid is perturbed by the presence of the acceptor vesicle.

Here we outline a novel assay to determine the transfer properties of charged lipids between phospholipid membranes. The method presented here has a number of advantages over previously reported methods. The lipid that is transferred does not have to be radio or fluorescently labelled^12^, so we can determine its unmodified transport properties. Another important advantage is that we are able to measure the lipid transfer continuously including any early molecular events (potentially in the millisecond time domain) as well as any long term changes that may also take place. This avoids many of the experimentally complex sampling and vesicle separation procedures^18,19^.

**Figure 1:**
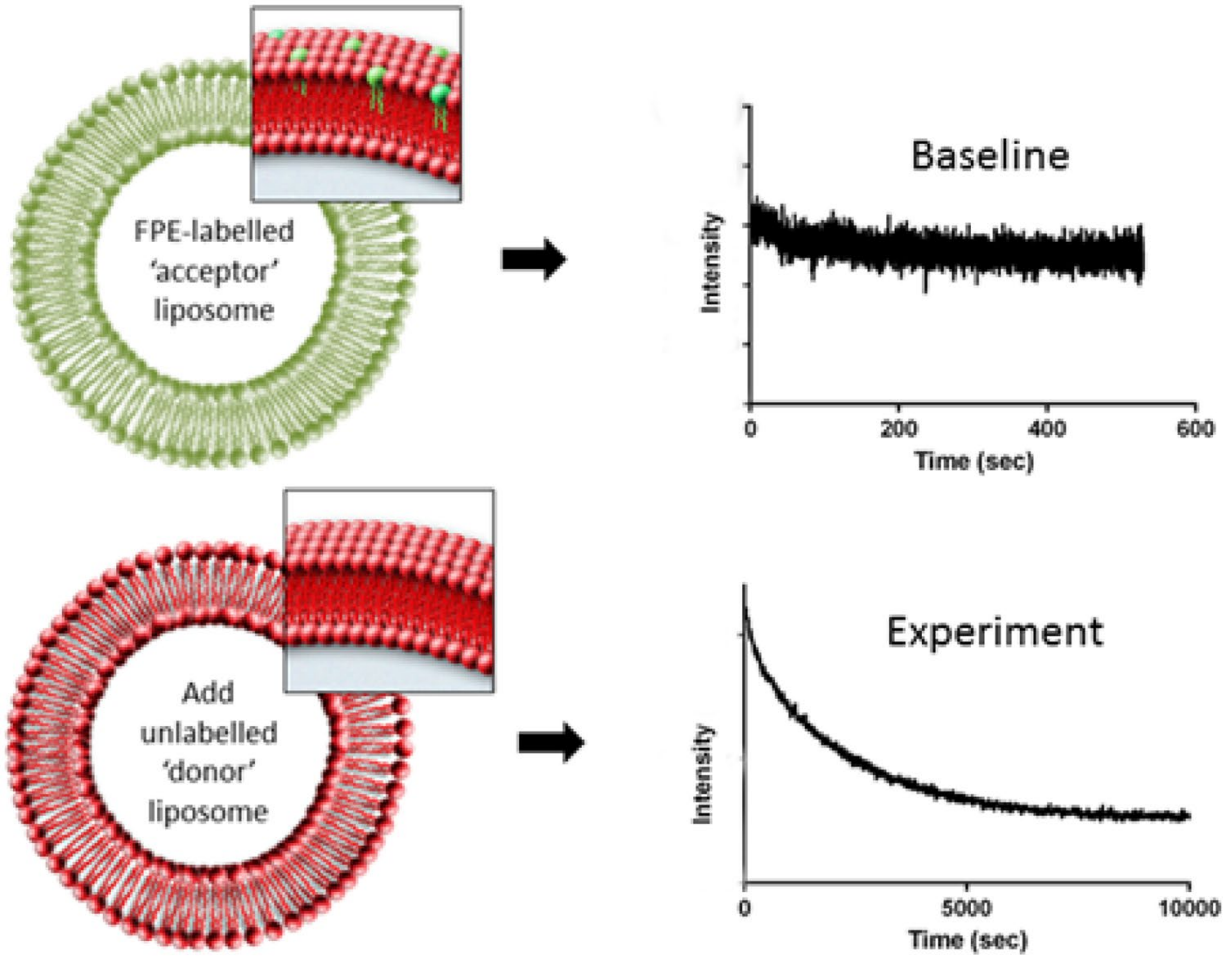
We use the well established donor/acceptor method to study lipid transfer, but unlike other implementations we label the neutrally charged acceptor vesicles with the fluorescent membrane probe FPE. Initially a stable baseline is established for the acceptor vesicles. When vesicles containing negative charges are added and are transferred, the fluorescent yield decreases and can be monitored over time.

The lipid transfer in the present study is detected via the modification of the electrostatic membrane surface potential of the acceptor vesicle membrane using a fluorescent membrane probe Fluoresceinphosphatidy-lethanolamine (FPE)^22^. Figure 1 illustrates the concept that we employ schematically. In the simplest terms the fluorescence yield of FPE reports the electrostatic surface potential of the membrane which is a function of the net excess surface charge density and the ambient ionic strength. At constant ionic strength, if electric charge is lost or added to a membrane containing FPE this leads to changes of the fluorescence yield. As FPE is known not to migrate from one membrane to another^23^ and the only charges that are present reside with the charged phospholipids, any fluorescence changes that occur act as a direct measure of the movement of the lipid species between vesicles.

The conceptual basis of the lipid-transfer measurement is analogous to studies we published previously in which we added free-fatty acids (FFA) to FPE-containing phospholipid vesicles^24^. Thus the addition of the FFAs to FPE-vesicles led to fluorescence changes that could be interpreted as binding and insertion of the FFA to the acceptor membrane. In this paper we demonstrate how the assay can be used to study the transfer properties of lipids with a systematic variation in head group and acyl chain length. The results of these experiment can help us to determine the underlying transfer mechanisms and their relevance to lipid transport that may feature to be important *in vivo*.

## Results

### Fluorescence-assay for detecting lipid exchange between phospholipid vesicles

**Figure 2:**
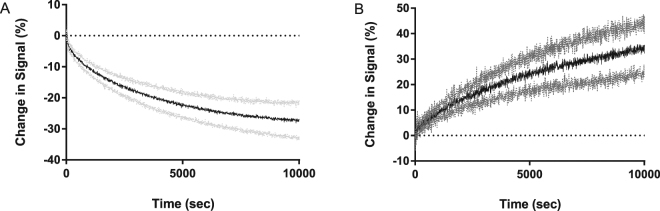
Binding interaction profiles of differentially charged SUV populations. (**A**) FPE-labelled DMPC100% SUVs plus unlabelled DMPC90%DMPS10% SUVs (**B**) FPE-labelled DMPC90%DMPS10% SUVs plus unlabelled DMPC100% SUVs. A stable fluorescence emission baseline was established for FPE-labelled SUVs (400 *μ*M) prior to the addition of unlabelled SUVs (400 *μ*M). In (**B**), the absolute signal intensity is reduced due to the presence of DMPS, therefore the signal noise appears to be greater when plotted as a % change.

Interactions between the phospholipids of distinct vesicle populations were monitored using FPE^22^. The origin of the fluorescence signal changes is dependent upon the nature and density of the net electrical charges located on the molecules that are and become membrane-bound. Typically, addition of positive charge or the loss of negative charge elicits an increase of the fluorescence yield (and *vice versa*). As an example of the simplest case in which the FPE-membrane system is used to report molecular interactions with membranes, the addition of Ca^2+^ ions to a labelled vesicle preparation leads to adsorption of the cations to the membrane surface which is perceived by the FPE as an increase of the electropositiveity or decrease of the electronegativity and elicits an increase in fluorescence as detailed in ref.^22^. For our present purposes we have chosen to label an acceptor vesicle with FPE and leave the donor vesicle unlabelled. Thus the expectation is that the acceptor vesicle would receive negative charge in the form of the lipid and thus the observed fluorescence yield would decrease (Fig. 2A). Similar studies in which the donor vesicle was labelled lead to an increase of the fluorescence yield (Fig. 2B) as the negative charge is depleted by lipid transfer.

Thus it was feasible to develop an assay that exploits the fluorescence detection of changes of the surface potential due to the loss or gain of charged lipids. The current experimental protocol involves allowing the FPE-labelled acceptor SUVs to exhibit a stable baseline measurement (around 500 seconds) at which time an equimolar concentration of unlabelled, differentially charged donor SUVs were added. Changes in fluorescence signal are monitored over time as the lipid transfer between the vesicle populations approaches an equilibrium. Figure 2A illustrates the time evolution of the fluorescence signals originating from such an experiment. A decrease in fluorescence signal over time was obtained following addition of the unlabelled DMPC90%DMPS10% (i.e. overall anionic) to FPE-labelled DMPC100% (neutral). The reverse of this system whereby the anionically-charged SUVs were FPE-labelled whilst the neutral SUVs are unlabelled resulted in an increase in fluorescence signal over time as anticipated and shown in Fig. 2B.

### Zeta Potential Measurements

**Figure 3:**
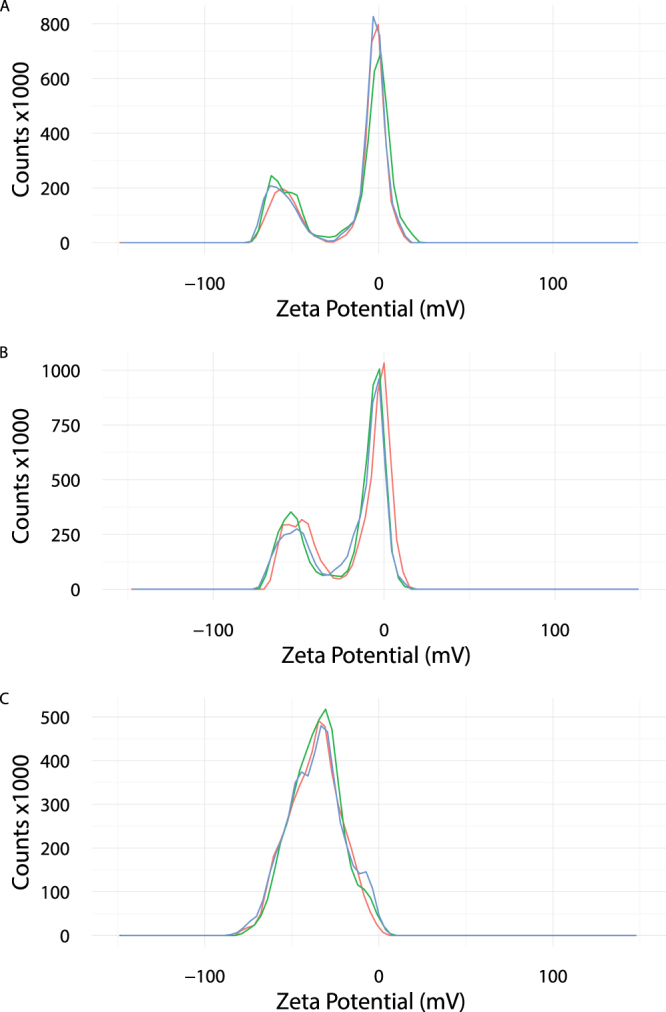
Zeta potential distributions of SUVs. Equimolar concentrations of unlabelled DMPC100% (400 *μ*M) and DMPC90%DMPS10% (400 *μ*M) were mixed and zeta potential measurements were recorded at (**A**) T = 0, (**B**) T = 6 hr and (**C**) T = 20 hr at room temperature using a Zetasizer Nano (Malvern Instruments, Malvern, UK). All measurements were performed in triplicate and plotted as coloured lines.

The output of the FPE-based assays provides a means of monitoring the lipid interactions and movement that occur between two vesicle populations. It is necessary, however, to validate that the data obtained is a consequence of transfer of phospholipids between SUVs rather than the transient movement of charged phospholipids into the proximity of the FPE sensor. Zeta potential measurements were used to define distinct populations of SUVs with and without anionic phospholipid content (Fig. 3A). Vesicles comprising DMPC90%DMPS10% and DMPC100% were found to have zeta potentials of −55 mV and −2.2 mV respectively. Upon mixing it was possible to monitor changes in the charge profiles of these populations as anionic phospholipid exchanged between the donor (DMPC90%DMPS10%) SUVs and acceptor (DMPC100%) SUVs. This phospholipid movement occurred over a period of several hours when monitored at room temperature. Within 20 hr the charge distribution appears to be homogeneous as one population with an average zeta potential of −36 mV was observed (Fig. 3B).

### The effect of the nature of the phospholipid head group and fatty acyl chain structure on the vesicle-vesicle membrane transfer properties

**Figure 4:**
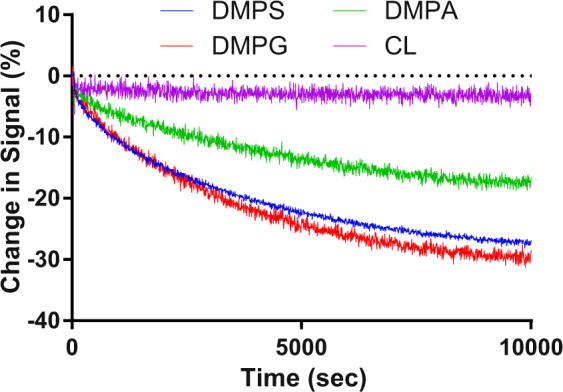
The effect of phospholipid head group on SUV binding interactions. A stable fluorescence emission baseline was established for FPE-labelled DMPC100% SUVs (400 *μ*M) prior to the addition of unlabelled DMPC90%DMPS10%, DMPC90%DMPG10%, DMPC90%DMPA10% or DMPC90%CL10% SUVs (400 *μ*M).

**Figure 5:**
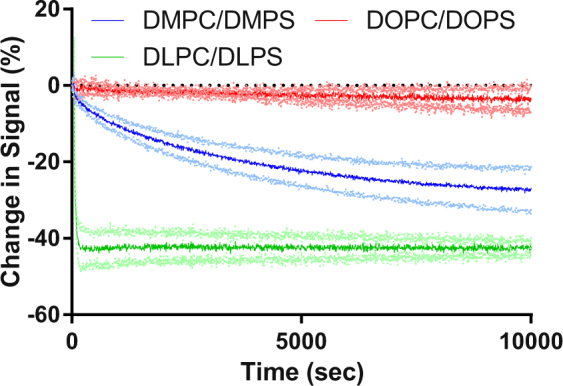
The effect of phospholipid acyl chain on SUV binding interactions. A stable fluorescence emission baseline was established for FPE-labelled PC SUVs (400 *μ*M DMPC100%, DLPC100% or DOPC100%) prior to the addition of the respective unlabelled anionically-charged SUV (400 *μ*M DMPC90%DMPS10%, DLPC90%DLPS10% or DOPC90%DOPS10%).

Further studies were undertaken to explore the effect of phospholipid head group charge and acyl chain structure on the transfer rate. The assay validation described above was undertaken using effectively neutrally charged DMPC100% and negatively charged DMPC90% DMPS10% SUVs. By varying the nature of the charge on the phospholipid head group and the structure of the acyl chain the effect of these factors on lipid transfer could be investigated. At pH7.4 the phospholipid head groups of DMPS, DMPG, DMPA and CL all possess negative charge. Data were collected as in Figs 4 and 5, showing the effect of headgroup and chain length on the charged lipid transfer rates. These kinetic changes were then fitted to a number of equations that represent different physical models.

## Analysis

A number of physical models have been considered for lipid transfer between membranes and we analyse our data in light of these models. There are perhaps two very different conceptual possibilities that may describe the lipid transfer. The simplest is phospholipids may adopt equilibria between the membrane vesicle and the aqueous bulk phase. Thus the individual phospholipids may then find their way back to a membrane from the water phase but not necessarily to the one they originated from. This would then allow easy transfer between membranes. A second, quite different mechanism involves collision between membrane vesicles that then facilitates mass (phospholipid) exchange between the vesicles. For our present purposes we find that the kinetic model which fits more closely to our data is closer to the first model and is described mathematically below. The physical interpretation of this model in which we also consider some modifications necessary to the molecular mechanism is outlined in the Discussion section.

Consider the following simple model where lipids can be exchanged via the aqueous medium^19^,

1 $$[{{\rm{C}}}_{d}]\underset{{{\rm{k}}}_{2}}{\overset{{{\rm{k}}}_{1}}{\rightleftharpoons }}[{{\rm{C}}}_{m}]\underset{{{\rm{k}}}_{4}}{\overset{{{\rm{k}}}_{3}}{\rightleftharpoons }}[{{\rm{C}}}_{a}]$$

where [*C*_*d*_] is the concentration of charged lipid in the donor vesicles, [*C*_*a*_] is the concentration of charged lipid in the acceptor vesicles, and [*C*_*m*_] is the concentration of charged lipid in the aqueous phase.

From this first order, two stage reversible reaction model we can define a set of differential equations for the charged lipid transfer rates,

2 $$\frac{d[{C}_{d}]}{dt}={k}_{2}[{C}_{m}]-{k}_{1}[{C}_{d}]$$

3 $$\frac{d[{C}_{a}]}{dt}={k}_{3}[{C}_{m}]-{k}_{4}[{C}_{a}]$$

4 $$\frac{d[{C}_{m}]}{dt}=-{k}_{2}[{C}_{m}]+{k}_{1}[{C}_{d}]-{k}_{3}[{C}_{m}]+{k}_{4}[{C}_{a}].$$

With the initial conditions *C*_*a*_(0) = 0, *C*_*m*_(0) = 0, *C*_*d*_(0) = 1, we can derive an analytical solution for the differential equations. The most general solution contains two exponential terms and a constant term. To describe the features of this solution we initially make the assumption that the phospholipid on (and off) rates for donors and acceptor vesicles are equal. In other words there is no difference between the interactions of the lipid monomers with the charged or uncharged vesicles. We set the on rates *k*_2_ = *k*_3_ = *k*_*n*_ and off rates *k*_1_ = *k*_4_ = *k*_*f*_ leading to,

5 $${C}_{d}(t)={\gamma }_{1}+\frac{1}{2}\,\exp \,(-{k}_{f}t)+\frac{{\gamma }_{2}}{2}\,\exp \,(-({k}_{f}+2{k}_{n})t)$$

6 $${C}_{a}(t)={\gamma }_{1}-\frac{1}{2}\,\exp \,(-{k}_{f}t)+\frac{{\gamma }_{2}}{2}\,\exp \,(-({k}_{f}+2{k}_{n})t)$$

7 $${C}_{m}(t)={\gamma }_{2}\,(1-\exp \,(-({k}_{f}+2{k}_{n})t)$$

where,

8 $${\gamma }_{1}=\frac{{k}_{n}}{{k}_{f}+2{k}_{n}}$$

9 $${\gamma }_{2}=\frac{{k}_{f}}{{k}_{f}+2{k}_{n}}$$

The steady state values for donor and acceptor are, *C*_*a*_ = *C*_*d*_ = *γ*_1_ and in the solution phase *C*_*m*_ = *γ*_2_. From previous studies we expect the off rate to be lower than the on rate, if significantly so, the donor and acceptors charged lipid component will approach 0.5 and the solution concentration will be very low. In other words, the charged lipid is equally divided between the donor and acceptor vesicle populations. By inspection of the ODE system above and also with numerical solutions we see that when *k*_*f*_/*k*_*n*_ is small, then a single exponential term is sufficient to determine the off rate but the on rate cannot be accurately determined.

The experimental fluorescence data acquired under the present circumstances contains an unknown scaling parameter linking % change in the FPE signal and the fractional charge transferred between vesicle populations. From these experiments we cannot know how the % change measured corresponds to charge fraction, as the FPE system can only be used in a differential manner, due to the unknown absolute quantity of FPE in each preparation of acceptor vesicles. It is feasible to calibrate the FPE-measurement system so absolute numbers of charges are determined by solution of the Poisson-Boltzmann equation^25,26^ and its relationship to the fluorescence yield.

We can see from Fig. 2 that by observing the amount of charge leaving an FPE labelled donor 2B, the % change in FPE signal is close to the reversed experiment where the acceptor is labelled. This supports the idea that after lipid transfer is completed, the charged lipid is evenly distributed between the donor and acceptor vesicles. We can use this assumption to inform the fitting process so that the rates can be approximated. This is done by adding a further parameter, a, which converts between the negative final steady state value and the expected charge fraction of 0.5. The fitting equation can be expressed as,

10 $${\rm{\Delta }}I=\frac{1}{2}a\,(1-\exp \,(-{k}_{f}t))$$

where Δ*I* is the change in fluorescence signal, *a* is the scaling factor relating charge fraction and signal changes, *k*_*f*_ is the off rate for charged lipids from the donor to acceptor.

**Figure 6:**
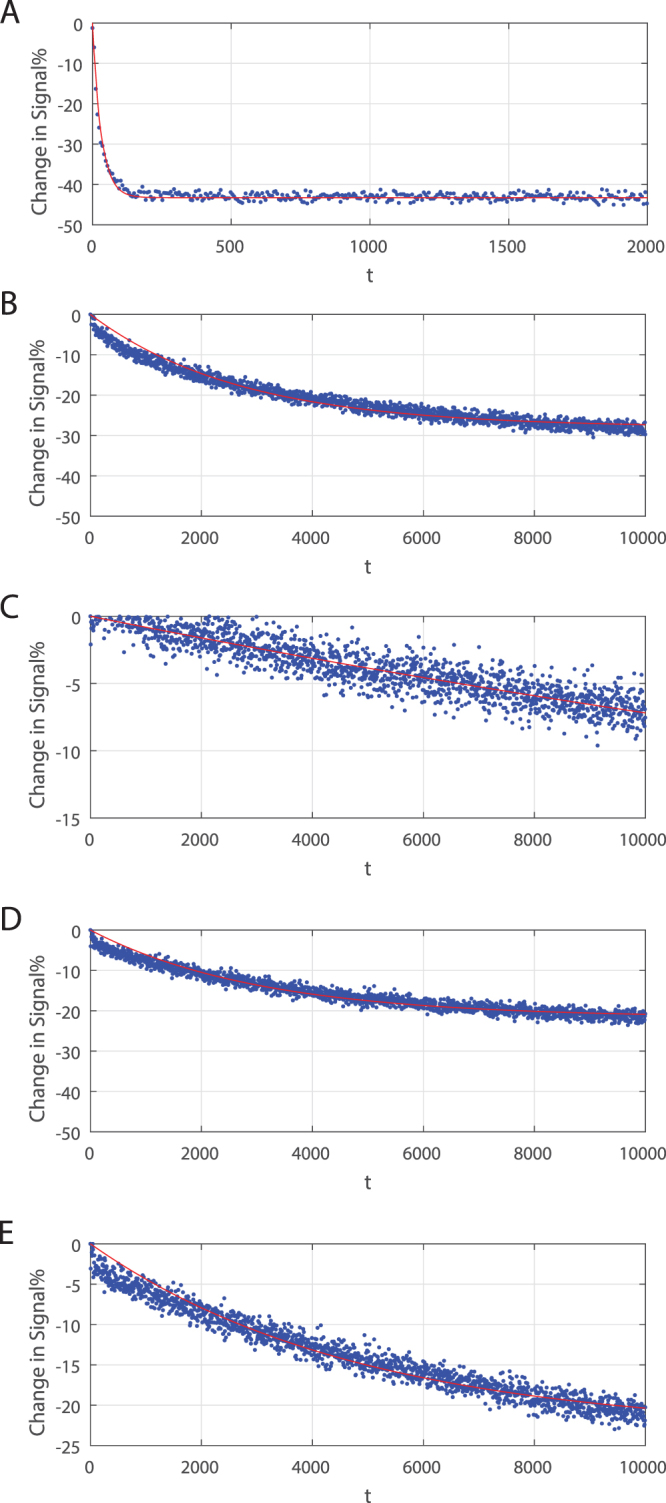
Fitting data with equation 10 where *γ*_2_ = 0 *γ*_1_ = 0.5 for lipid types (**A**) DLPC/DLPS, (**B**) DMPC/DMPS, (**C**) DOPC/DOPS, (**D**) DMPC/DMPG, (**E**) DMPC/DMPA.

Figure 6A shows a single exponential fit for the acceptor concentration *C*_*a*_ for DLPC/DLPS which has the highest rate of transfer. Parameters derived from these fits are summarised in Tables 1 and 2 for acyl chain and head group variations respectively. Cardiolipin transfer rate are not shown as they were too slow to be determined. Thus this appears to be a very slow process and may help understand the deeper molecular processes involved in the molecular mechanism of the transfer process. This will be explored in future work.

**Table 1:** Spontaneous transfer rates for lipids with different acyl chains.

Lipid Type	*a*	*k*_*f*_ (*s* ^−1^)
DLPC/DLPS	−85 ± 7	0.043 ± 0.012
DMPC/DMPS	−54 ± 11	39 × 10^−5^ ± 8.9 × 10^−5^
DOPC/DOPS	−58	2.9 × 10^−5^

**Table 2:** Spontaneous transfer rates for lipids with different head groups.

Lipid Type	*a*	*k*_*f*_ (*s* ^−1^)
DMPC/DMPG	−61 ± 15	34 × 10^−5^ ± 1.3 × 10^−5^
DMPC/DMPS	−54 ± 11	39 × 10^−5^ ± 8.9 × 10^−5^
DMPC/DMPA	−39 ± 9	30 × 10^−5^ ± 21 × 10^−5^

Observed deviations from the single exponential fits shown in Fig. 6 cannot be explained by an additional exponential term due to the on rate. Other factors could be asymmetry in the off rates for donors and acceptors due to initial differences in charge. These complexities point towards the possibility that charge dependent rates exist. These additional complexities will be explored in our future study.

## Discussion

Spontaneous transfer of phospholipids between membrane systems is often overlooked as a possible feature in biological systems as well as a factor in more simple artificial membrane systems. This seems to be due to the apparent consensus opinion that the process was thought likely to be too slow to be biologically relevant^27^. Based on our present study however, this may be an over simplification of a possibly much more complicated system. This has implications for the mechanisms of how biological systems may ‘handle’ their lipid trafficking requirements and will be discussed in more detail in the future. For the moment we consider the physical mechanisms of how the phospholipids may migrate from one membrane to another but for completeness we indicate how this may feature in biological systems.

Many studies have shown that at low vesicle concentrations lipids may be spontaneously transferred from a membrane via a first-order process along with diffusion through the aqueous phase from a donor membrane to an acceptor membrane. The desorption process of the lipid from the donor appears to limit the rate at which this can occur. A study by Nichols^15^ used the resonant energy transfer between fluorescently labelled lipids to propose an energy diagram for lipid monomer vesicle interactions. By studying the transfer of NBD labelled PC lipids with different chain lengths at different temperatures, a number of useful features were discovered. Namely, for monomer disassociation a high energy transition state forms, where the lipid emerges from the bilayer surface, creating a cavity within the bilayer and also in the water phase to accommodate the lipid molecule. Enthalpy appears to be a determinant of the creation of the high energy transition state. It was shown that increasing the chain length results in a higher activation energy for monomer disassociation and also lowers the free energy of transfer of monomers from water to vesicle.

The methodology we employ in the present study can be used to assess the effects on the rates of transfer of charged phospholipids by the presence of other types with the most dramatic effect exhibited by cardiolipin. By increasing the chain length, the transfer rate decreases which is consistent with the energy model proposed by Nichols^15^. We observe a difference in the transfer rates for charged lipids with different headgroups. We suggest that the sequential order that arises from our experimental data (Tables 1 and 2) is related to the relative activation energies to disassociate each lipid type from the bilayer, in an analogous way to the chain length dependence. At present we cannot point to a simple statement or experimental study that correlates to the order shown, only that computational calculations of the lipids used in this study show a range of headgroup intermolecular interactions with surrounding lipids that contribute to disassociation energy^28^. Our approach however, in which we derive the rates of transfer, may better validate or inform these models about the headgroup intermolecular interactions.

When vesicles interact, attractive van der Waals interactions bring vesicles into proximity. At this distance (around 1.5 nm) the formation of the activated state is enhanced by the proximity of the apposing vesicle^13,20^. An additional contribution to the increased transfer rate, could be due to the modification of the water structure between the two vesicles which will lower the solvation energy of the desorbed lipid^29^. Thus as the donor and potential acceptor membrane come into close proximity (ca. 3 nm) the movement of the phospholipid does not need to encounter bulk phase water which would involve greater energetic consequences, rather the much lower Born energy implications of the lipids moving in a lower dielectric constant media^29^ would allow an energetically easier ‘hop’ between respective membranes. It has been shown experimentally that only at high concentrations of vesicles are second order interactions seen, and calculations show that vesicle collision mediated transfer events are unlikely^20^. In addition to these findings, it has been shown that second order processes are also dependent on charge content and surface hydration^30^. By modifying and reducing the equilibrium separation distance by including 30%mol DMPE, the collision mediated transfer efficiency was increased 100 times^30^.

In our experiments at low vesicle concentrations and with highly charged vesicles we do not expect to observe second-order processes but our technique will enable the kinetics of these processes to be more easily determined at higher vesicle concentrations.

## Materials and Methods

### Materials

All the lipids were purchased from Avanti Polar Lipids (Alabama, USA) in lyophilized powder form at a purity of >99% so no further purification was necessary. The lipids used were 1,2-dilauroyl-sn-glycero-3-phospho-L-serine (DLPC), 1,2-dilauroyl-sn-glycero-3-phospho-L-serine (DLPS), 1,2-dimyristoyl-sn-glycero-3-phosphate (DMPA), 1,2-dimyristoyl-sn-glycero-3-phosphocholine (DMPC), 1,2-dimyristoyl-sn-glycero-3-phospho-(1′-rac-glycerol) (DMPG), 1,2-dimyristoyl-sn-glycero-3-phospho-L-serine (DMPS), 1,2-dioleoyl-sn-glycero-3-phosphocholine (DOPC), 1,2-dioleoyl-sn-glycero-3-phospho-L-serine (DOPS), 1,2-dipalmitoyl-sn-glycero-3-phosphocholine (DPPC), 1,2-dipalmitoyl-sn-glycero-3-phospho-L-serine (DPPS) and Cardiolipin (1′,3′-bis[1,2-dimyristoyl-sn-glycero-3-phospho]-sn-glycerol) (CL).

Fluoresceinphosphatidylethanolamine (FPE; F-362) was purchased from Life Technologies (Paisley, UK). All other reagents were supplied at the highest purity available by Sigma Aldrich (Poole, UK).

### Small unilamellar vesicle (SUV) preparation and labelling

Lipids were co-dissolved at the desired concentrations in chloroform, dried under a stream of oxygen-free nitrogen gas for 3 hours and placed in a vacuum for a minimum of 12 hours, after which they were sealed and stored at −20 °C before use. Samples were hydrated in 10 mM tris pH7.4 to a concentration of 13 mM. After hydration, each sample was heat cycled (between approximately −200 °C and 60 °C) a minimum of five times and extruded 21 times through a 25 mm diameter polycarbonate filter with pores of 100 nm in diameter (Nucleopore Corp.). SUVs were labelled in the outer bilayer leaflet with FPE as previously described^22^. Briefly, the SUVs were incubated with ethanolic-FPE (never more than 0.1% ethanol of the total aqueous volume) at 37 °C for 1.5 h in the dark. Unincorporated FPE was removed by gel filtration on a PD10 Sephadex column.

### Spectroscopy

Fluorescence spectroscopy was conducted on a FluoroMax-4 Spectrometer (HORIBA Jobin Yvon). Excitation and emission wavelengths were set at 490 and 518 nm respectively. A stable fluorescence intensity baseline was established for the FPE-labelled acceptor SUVs (400 *μ*M) after which point an equimolar amount of unlabelled donor SUVs was added. Fluorescence changes versus time were recorded at 37 °C. Experimental controls comprised subtraction of the fluorescence signal obtained following mixing of FPE-labelled and unlabelled SUVs of identical phospholipid composition. For all experiments shown here data displayed is a percentage change of the initial signal (mean ± SD) for at least 3 repeat experiments.

### Zeta potential measurements

Equimolar concentrations of DMPC100% and DMPC90%DMPS10% vesicles were mixed and incubated at room temperature. Zeta potential measurements were recorded prior to mixing, immediately following mixing and then at 3, 6 and 20 hr timepoints using a Zetasizer Nano (Malvern Instruments, Malvern, UK). All measurements were performed in triplicate.

### Modelling and Fitting

Data aggregation and plotting was done with Graphpad Prism. Fitting was carried out with the MATLAB curve fitting tool using the Trust-Region algorithm. Model evaluation was done using MATLAB using the ODE solvers in addition to the muPad package.

### Data availability

The datasets generated during and/or analysed during the current study are available from the corresponding author on reasonable request.
